# Pharmacogenetic determinants of tenofovir diphosphate and lamivudine triphosphate concentrations in people with HIV/HBV coinfection

**DOI:** 10.1128/aac.00549-24

**Published:** 2024-07-30

**Authors:** Jihyun Bae, Marwa Tantawy, Yan Gong, Taimour Langaee, Margaret Lartey, Vincent Ganu, Kenneth Tachi, Oluwayemisi Ojewale, Adjoa Obo-Akwa, Isaac Boamah, Lane R. Bushman, Lucas Ellison, Hongmei Yang, Peter L. Anderson, Awewura Kwara

**Affiliations:** 1 Center for Pharmacogenomics and Precision Medicine, College of Pharmacy, University of Florida, Gainesville, Florida, USA; 2 Department of Medicine, Korle-Bu Teaching Hospital, Accra, Ghana; 3 Department of Medicine and Therapeutics, University of Ghana Medical School, College of Health Sciences, University of Ghana, Accra, Ghana; 4 Department of Medicine, Division of Infectious Diseases and Global Medicine, University of Florida, Gainesville, Florida, USA; 5 Colorado Antiviral Pharmacology Laboratory and Department of Pharmaceutical Sciences, Skaggs School of Pharmacy and Pharmaceutical Sciences, University of Colorado-Anschutz Medical Campus, Aurora, Colorado, USA; 6 Department of Biostatistics and Computational Biology, University of Rochester School of Medicine and Dentistry, Rochester, New York, USA; 7 Medical Service, North Florida South Georgia Veterans Health System, Gainesville, Florida, USA; Providence Portland Medical Center, Portland, Oregon, USA

**Keywords:** pharmacogenetics, hepatitis B virus, TFV-DP, human immunodeficiency virus, 3TC-TP

## Abstract

The nucleos(t)ide analogs require phosphorylation to the pharmacologically active anabolites in cells. We investigated the hypothesis that single-nucleotide polymorphisms (SNPs) in genes that encode transporters and phosphodiesterase (PDE) enzymes involved in tenofovir (TFV), disoproxil fumarate (TDF), and lamivudine (3TC) disposition will be associated with concentrations of their phosphate anabolites and virologic response. Individuals with human immunodeficiency virus (HIV) and hepatitis B virus (HBV) coinfection receiving TDF/3TC-containing antiretroviral therapy were enrolled. Steady-state TFV diphosphate (TFV-DP) and 3TC triphosphate (3TC-TP) concentrations in peripheral blood mononuclear cells (PBMCs) and dried blood spot samples were quantified. The relationship between genetic variants and TFV-DP and 3TC-TP concentrations as well as with virologic response were examined using multivariable linear regression. Of the 136 participants (median age 43 years; 63% females), 6.6% had HBV non-suppression, and 7.4% had HIV non-suppression. The multidrug resistance protein 2 (encoded by *ABCC2* rs2273697) SNP was associated with 3TC-TP concentrations in PBMCs. The human organic anion transporter-1 (encoded by *SLC28A2*) rs11854484 SNP was associated with HIV non-suppression, and when evaluated together with SNPs with marginal associations (*ABCC2* rs717620 and *PDE1C* rs30561), participants with two or three variants compared to those with none of these variants had an adjusted odds ratio of 48.3 (confidence interval, 4.3–547.8) for HIV non-suppression. None of the SNPs were associated with HBV non-suppression. Our study identified *ABCC2* SNP to be associated with 3TC-TP concentrations in PBMCs. Also, a combination of genetic variants of drug transporters and PDE was associated with HIV non-suppression.

## INTRODUCTION

Human immunodeficiency virus (HIV) and hepatitis B virus (HBV) coinfection is common because of the shared routes of transmission. In 2015, about 2.7 million (7.4%) of the 36.7 million people with HIV (PWH) worldwide were infected with HBV, with 71% (1.96 million) living in sub-Saharan Africa (1). Ongoing HBV replication in individuals with HIV/HBV coinfection poses a higher risk of complications such as liver cirrhosis, hepatocellular carcinoma, and liver-related death compared to those without HIV (2–6). Thus, the goal of antiretroviral therapy (ART) in individuals with coinfection is sustained suppression of both HIV and HBV. The nucleos(t)ide analogs (NAs), tenofovir (TFV), disoproxil fumarate (TDF), TFV alafenamide (TAF), emtricitabine (FTC), and lamivudine (3TC) are potent inhibitors of HIV reverse transcriptase and HBV DNA polymerase. Thus, the World Health Organization guidelines recommend a combination of TDF (or TAF) plus 3TC (or FTC) as essential components of ART regimens for people with HIV/HBV coinfection (7). In resource-limited settings where TAF and FTC are not readily available, TDF/3TC is the nucleoside backbone of recommended ART regimens in people with HIV/HBV coinfection (8).

Despite the high barrier to TFV resistance, incomplete suppression of HBV replication on TDF-containing ART is common (9–16). The activity of TDF and 3TC is dependent on the concentrations of the active phosphate anabolites, TFV diphosphate (TFV-DP), and 3TC triphosphate (3TC-TP), respectively (17–19). Upon absorption, TDF is thought to be rapidly converted by a carboxylesterase to a monoester and then to TFV by a phosphodiesterase (PDE) upon first pass through the liver (19). The uptake of TFV into cells is mediated by organic anion transporters 1 and 3 (OAT1 and OAT3) and efflux by multidrug resistance-associated proteins (MRP) 1, 4, and 5 (19). 3TC is transported into cells by organic cation transporters 1 and 2 (OCT1 and OCT2) (20) and may be a substrate of the concentrative nucleoside transporter 1 (CNT1) (21). The efflux transporters MRP4 encoded by *ABCC4* play an important role in the transport of the 3TC out of cells (22, 23), but the role of MRP2 in the transport of 3TC or its anabolites is not well established (23). We hypothesize that single-nucleotide polymorphisms (SNPs) in genes that encode uptake transporters, efflux transporters, and/or PDE enzymes would be associated with the TFV-DP and/or 3TC-TP concentrations, as well as with virologic response in individuals with HIV/HBV coinfection on TDF/3TC-containing ART.

## MATERIALS AND METHODS

### Study participants

A cross-sectional pharmacokinetic (PK) and PK/pharmacodynamic (PD) study was performed at the Korle-Bu Teaching Hospital (KBTH) in Ghana from 9 November 2020 to 17 November 2021, as we previously reported (24). People with HIV infection aged 18 years or older with a positive hepatitis B surface antigen on TDF/3(F)TC-containing ART for at least 9 months were enrolled. Individuals with HIV-2 or HIV-1/2 dual infection were excluded. Participants were scheduled for a study visit where demographic and clinical data were collected using standardized forms. Self-reported adherence using the five-point Likert scale, which employs a 7-day recall, was used to assess the level of adherence as it is routinely used in our clinic. Plasma HBV DNA and HIV RNA quantification was performed by an international organization for standardization (ISO)-certified commercial laboratory service provider in Ghana as previously reported (24). The lower limit of detection of the real-time Cobas assay for HIV-1 RNA is 20 copies/mL, with a linear range of 20–10,000,000 copies/mL. HIV RNA ≥20 copies/mL was considered non-suppressed. The lower limit of detection of the Roche assay for HBV DNA is 20 IU/mL, and the linear range is 20–170,000,000 IU/mL. HBV DNA ≥20 IU/mL was considered non-suppressed.

### Pharmacokinetic testing and analysis

The details of PK testing and analysis were previously reported (24). At the study visit, 4 mL of blood was collected in EDTA vacutainers to spot dried blood spots and isolate peripheral blood mononuclear cells (PBMCs). Twenty-five microliters of whole blood was transferred to a Whatman 903 card using a pipette, and the cards were air-dried for at least 3 hours at room temperature prior to storage at −80°C. Standard centrifugation and Ficoll separation procedures were used to isolate PBMCs, which were then counted and lysed according to the established protocols (25). Frozen samples were shipped to the University of Colorado Antiviral Pharmacology Laboratory, and the steady-state concentrations of TFV-DP and 3TC-TP were quantified from PBMCs and a 3 mm punch using a previously validated method (24). Concentrations of TFV-DP and 3TC-TP below the lower limit of quantitation (LLOQ), which were 25 fmol/sample and 100 pmol/sample, respectively, were assigned a value half of the LLOQ for analysis to avoid counting the participants with below lower limit of quantitation (BLLOQ) as missing as done in other studies (26, 27). The concentrations of TFV-DP and 3TC-TP in PBMCs were used as the reference for the active site. As we previously reported, given the unexplained lower concentrations of TFV-DP in dried blood spots (DBSs) in the study population compared to expected values (described below), TFV-DP in PBMCs (fmol/10^6^ cells) was used to categorize adherence (PK-determined adherence) as follows: ≥52 for seven doses per week, 30–51 for four to six doses per week, 15–29 for two to three doses per week, and <15 for less than two doses per week (24).

### DNA isolation and genotyping

Genomic DNA was isolated from blood lymphocytes using a commercially available kit (QIAamp DNA Blood Mini Kit, Qiagen Inc., California, USA). Genotyping for SNPs in MDR1 (encoded by *ABCB1*)*,* MRP2 (*ABCC2*)*,* MRP4 (*ABCC4*), MRP7 (*ABCC10*), *PDE1C, PDE3A, PDE4D, PDE11A*, and *SLC28A2* [which encodes the influx transporter, human organic anion transporter-1 (hOAT1)] was performed by TaqMan allelic discrimination using the fluorescence-based TaqMan Quant Studio Real-Time PCR System according to the manufacturer’s recommendations (Life Technologies/Fisher Scientific, Foster City, CA). We selected SNPs in *ABCB1*, *SCL22A6*, *ABCC2*, and *ABCC4* with plausible effects on TFV and/or 3TC transport that may influence cellular influx or efflux of the NAs. Genes encoding PDE enzyme SNPs were included as they may influence TDF disposition. Specifically, *ABCC4* 1612C>T rs1557070, *ABCC4* 3463G>A rs1751034, *ABCC4* 3724G>A rs11568695, and *ABCC4* 4131T>G rs3742106 SNPs were selected as candidate SNPs since they have been putatively associated with intracellular NA phosphate anabolite concentrations in prior studies (28–30).

### Statistical analysis

The demographic characteristics of the participants were presented as medians and interquartile ranges (IQRs) for continuous variables and the frequency and percentage for categorical variables. The allele frequencies of all SNPs were calculated. Hardy–Weinberg equilibrium (HWE) was tested using the chi-squared test with one degree of freedom. Associations between SNPs and steady-state intracellular concentrations of TFV-DP and 3TC-TP were analyzed by using univariate and multivariable linear regression adjusted for age, gender, body mass index (BMI), and CD4 count. Multivariable logistic regression was also performed to estimate the odds ratio (OR) and 95% confidence interval (CI) of unsuppressed HBV DNA and HIV RNA. An additive mode of inheritance was used for all SNPs except for the SNPs with low minor allele frequencies where we used the dominant mode of inheritance. SNPs with *P* < 0.05 were considered statistically significant. All analyses were performed using SAS v 9.4 (Cary, NC).

## RESULTS

### Study population

During the study period, 138 eligible individuals were enrolled, of whom two were excluded from the current analysis due to poor sample quality and inaccurate drug concentration due to an error in PBMC cell count. Of the 136 participants in the final analysis (Table 1), 86 (63%) were female, the median (IQR) age was 43.0 (12.0) years, and CD4 count was 468.5 (315.0) cells/µL. The median (IQR) duration of TDF/3TC-containing ART was 7.0 (4.0) years.

**TABLE 1:** Baseline characteristics of HIV/HBV coinfected participants treated with TDF/3TC-containing antiretroviral therapy (*N* = 136)^*a*^

Characteristic	Number (%) or median (IQR)
Age (years)	43.0 (12.0)
BMI (kg/m^2^)	26.1 (7.9)
CD4 count (cells/µL)	468.5 (315.0)
Duration of antiretroviral therapy (years)	7.0 (4.0)
Concentration of antiretroviral therapy	
TFV-DP in PBMCs (fmol/10^6^ cells)	104.1 (87.4)
3TC-TP in PBMCs (pmol/10^6^ cells)	10.3 (6.7)
TFV-DP in DBS (fmol/punch)	417.2 (344.0)
3TC-TP in DBS (pmol/punch)	0.19 (0.11)
Sex	
Female	86 (63.2%)
Male	50 (36.8%)
HBe antigen positive	4 (2.9%)
HBe antibody positive	42 (30.4%)
ART	
TDF/3TC/dolutegravir	115 (84.6%)
TDF/3TC/efavirenz	21 (15.4%)
Ever forgotten to take medications?	
Yes	17 (12.5%)
No	119 (87.5%)
HIV suppressed	
Yes	126 (92.6%)
No	10 (7.4%)
HBV suppressed	
Yes	127 (93.4%)
No	9 (6.6%)
HIV RNA level (copies/mL)	
<20	128 (92.8%)
20–<200	1 (0.7%)
200–<1,000	4 (2.9%)
>1,000	5 (3.6%)
HBV DNA (IU/mL)	
<20 IU/mL	129 (93.5%)
20–2,000	4 (2.9%)
2,001–20,000	2 (1.4%)
>20,000	3 (2.2%)

^*a*^ For continuous variables, medians (IQR) are reported.

Nine participants (6.6%) had unsuppressed HBV DNA, 10 (7.4%) had unsuppressed HIV RNA, and 6 had suppressed HIV RNA but unsuppressed HBV DNA. As previously reported in the parent study (24), participants with unsuppressed HBV had lower median (IQR) TFV-DP concentration in PBMC [71.7 (55.0–88.6) vs 109 (63.2–153.7) fmol/10^6^ cells, *P* = 0.049] and DBS [241 (186–343) vs 444.1 (295.9–634.8) fmol/punch, *P* = 0.0029] compared to those with suppressed HBV. Overall, 128 (92.8%) reported no missed ART doses in the week prior to study visits, but based on TFV-DP concentration in PBMCs, 114 (83.2%) had concentration commensurate with seven doses per week (24).

Overall, 18 SNPs were genotyped, 1 SNP (*PDE4D* rs1546221) was monomorphic, and 2 SNPs (*ABCC10* rs9349256 and *ABCC4* rs1059751) deviated from HWE and were excluded from further analysis. The frequencies of genetic polymorphisms of the 15 evaluated SNPs are shown in Table 2.

**TABLE 2:** The minor allele frequencies and Hardy–Weinberg equilibrium test of the 15 SNPs^*a*^

SNPs	Minor/major alleles	Minor allele frequency	HWE test (*P*)
*ABCB1* rs1045642	A/G	0.11	0.52
*ABCC2* rs17216177	C/T	0.21	0.94
*ABCC2* rs2273697	A/G	0.16	0.85
*ABCC2* rs3740066	T/C	0.26	0.17
*ABCC2* rs717620	T/C	0.04	0.62
*ABCC4* rs1751034	C/T	0.24	0.66
*ABCC4* rs11568695	T/C	0.20	0.70
*ABCC4* rs3742106	C/A	0.24	0.12
*ABCC4* rs1557070	A/G	0.32	0.57
*PDE1C* rs30561	T/C	0.11	0.63
*PDE3A* rs11045347	T/C	0.43	0.96
*PDE4D* rs37576	C/T	0.31	0.84
*PDE4D* rs6889641	A/G	0.24	0.62
*PDE11A* rs2695735	G/A	0.08	0.33
*SLC28A2* rs11854484	T/C	0.09	0.96

^*a*^ *ABCB,* adenosine triphosphate (ATP)-binding cassette subfamily B; *ABCC,* ATP-binding cassette subfamily C; *SLC,* solute carrier family.

### Association of SNPs and TFV-DP and 3TC-TP concentrations

**Fig 1:**
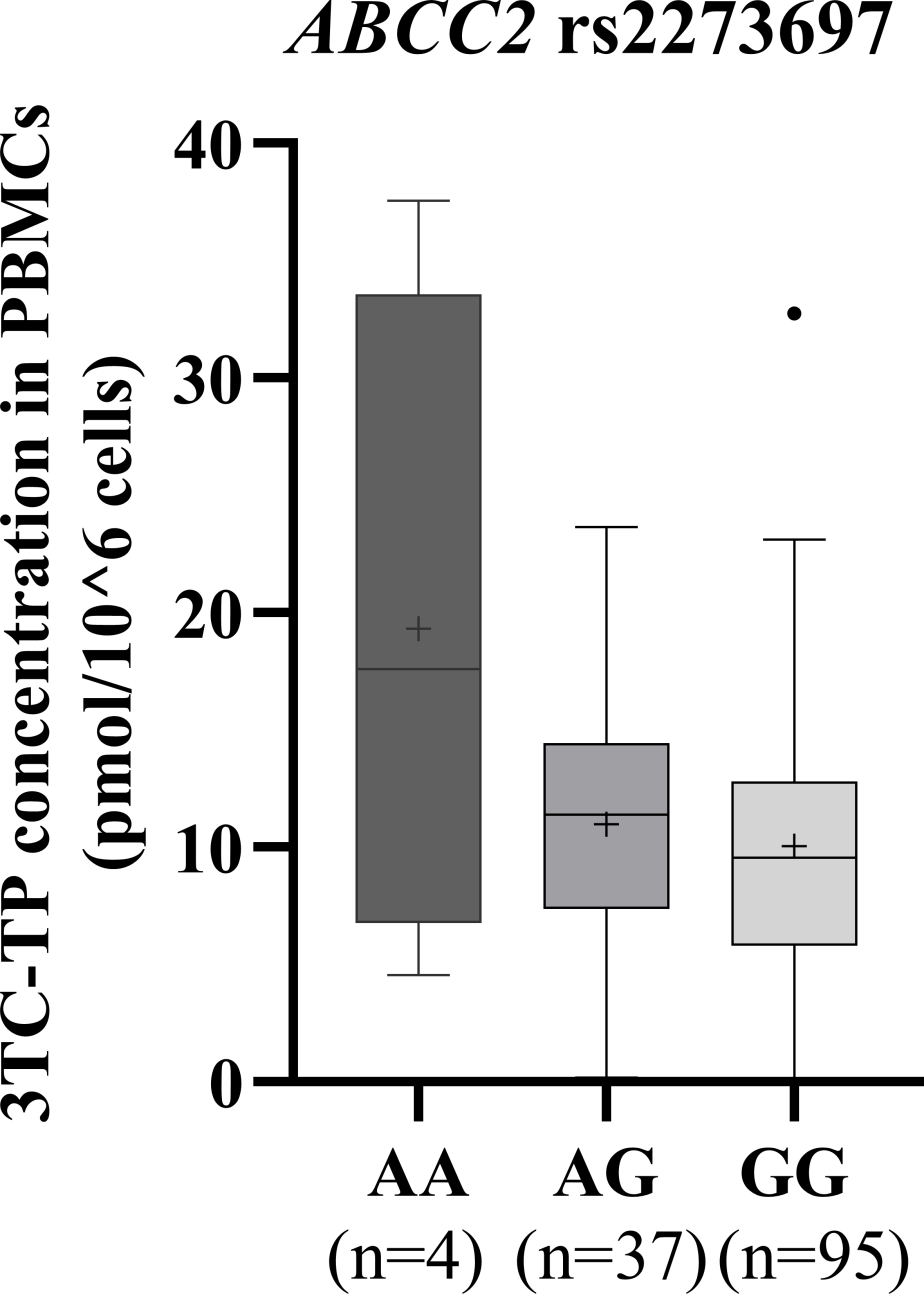
Box plot of 3TC-TP concentrations in peripheral mononuclear cells by the ATP-binding cassette sub-family C member 2 (*ABCC2*) rs2273697 genotype. The top of the box represents the 75th percentile (Q3), and the bottom of the box represents the 25th percentile (Q1). The horizontal line in the middle of the box represents the median value, and the “+” represents the mean value. The top whisker represents the maximum or Q3 + 1.5*IQR, whichever is lower. The bottom whisker represents the minimum or Q1-1.5*IQR, whichever is higher. The dots represent outliers. The unadjusted *P* = 0.032, and the adjusted *P* = 0.015.

The median TFV-DP and 3TC-TP concentrations by genotypes are presented in Table 3. In unadjusted and adjusted analyses, the variant allele A of *ABCC2* rs2273697 SNP was associated with higher intracellular 3TC-TP concentrations in PBMC, with median concentrations of 17.57, 11.40, and 9.56 pmol/10^6^ cells in participants with AA, AG, and GG genotypes, respectively (unadjusted *P* = 0.032 and adjusted *P* = 0.015) (Fig. 1). No other associations were significant after adjusting for covariates. In unadjusted analysis, *PDE4D* rs6889641 (*P* = 0.028) and *ABCC2* rs17216177 (*P* = 0.049) were associated with TFV-DP and 3TC-TP in PBMCs, respectively, while *PDE1C* rs30561 (*P* = 0.038) and *ABCC2* rs3740066 (*P* = 0.0034) were associated with TFV-DP in DBS. The variant allele T carriers (CT/TT) of *SLC28A2* rs11854484 had marginally lower levels of 3TC-TP in DBS (*P* = 0.071) compared to those with CC genotype in the unadjusted analysis (Table 3).

**TABLE 3:** Median (interquartile range) of TFV-DP and 3TC-TP concentrations in PBMCs and DBS by genotype^*a*^

SNP	PBMC, median (interquartile range)		DBS, median (interquartile range)	
	TFV-DP (fmol/10^6^ cells)	3TC-TP (pmol/10^6^ cells)	TFV-DP (fmol/punch)	3TC-TP (pmol/punch)
*ABCB1* rs1045642				
GG (*N* = 106)	106.78 (90.65)	10.33 (7.03)	418.70 (340.26)	0.19 (0.11)
AG/AA (*N* = 30)	90.13 (78.84)	10.36 (4.29)	391.21 (326.84)	0.20 (0.12)
*ABCC2* rs17216177		^*^P = 0.049/*P*_adj_ = 0.065		
TT (*N* = 85)	109.14 (94.90)	11.40 (6.85)	445.00 (366.39)	0.19 (0.10)
CT (*N* = 45)	96.32 (80.42)	9.08 (8.00)	401.74 (343.88)	0.19 (0.13)
CC (*N* = 6)	107.96 (93.21)	10.01 (5.30)	285.94 (351.38)	0.17 (0.14)
*ABCC2* rs2273697		^*^P = 0.032/^**^P_adj_ = 0.015		
GG (*N* = 94)	103.17 (92.12)	9.56 (6.97)	423.55 (317.03)	0.18 (0.11)
AG (*N* = 37)	108.04 (73.96)	11.40 (6.33)	416.05 (202.27)	0.20 (0.12)
AA (*N* = 4)	138.17 (78.53)	17.57 (20.54)	486.10 (326.39)	0.26 (0.13)
*ABCC2* rs3740066			^*^P = 0.0034	
CC (*N* = 79)	104.48 (82.04)	10.53 (7.70)	389.67 (267.17)	0.18 (0.10)
CT (*N* = 45)	96.32 (98.48)	9.50 (6.26)	418.71 (302.12)	0.19 (0.11)
TT (*N* = 12)	120.49 (102.68)	11.76 (5.87)	672.37 (290.53)	0.24 (0.15)
*ABCC2* rs717620				
CC (*N* = 125)	108.04 (88.69)	10.20 (6.90)	417.97 (336.92)	0.19 (0.11)
CT (*N* = 11)	67.37 (62.07)	12.22 (6.57)	374.24 (433.38)	0.17 (0.28)
*ABCC4* rs1751034	*P* = 0.068			
TT (*N* = 78)	114.88 (90.65)	10.19 (6.26)	391.37 (333.94)	0.19 (0.11)
CT (*N* = 52)	94.06 (83.58)	10.72 (8.11)	432.44 (378.69)	0.19 (0.12)
CC (*N* = 6)	97.99 (105.51)	10.05 (3.85)	533.87 (173.62)	0.18 (0.02)
*ABCC4* rs11568695				
CC (*N* = 85)	103.17 (81.90)	10.18 (7.38)	438.95 (321.01)	0.18 (0.11)
CT (*N* = 46)	99.10 (83.02)	10.79 (5.21)	381.27 (422.73)	0.19 (0.12)
TT (*N* = 5)	176.12 (64.85)	13.86 (11.32)	560.45 (183.25)	0.18 (0.08)
*ABCC4* rs3742106				
AA (*N* = 83)	103.17 (82.04)	10.53 (8.24)	418.71 (336.60)	0.19 (0.10)
AC (*N* = 42)	110.95 (97.32)	10.23 (5.71)	391.37 (379.46)	0.17 (0.13)
CC (*N* = 11)	109.14 (82.91)	10.39 (8.02)	374.24 (254.44)	0.17 (0.28)
*ABCC4* rs1557070				
GG (*N* = 61)	95.49 (74.02)	10.27 (5.47)	391.37 (282.23)	0.19 (0.12)
AG (*N* = 62)	113.43 (93.19)	10.72 (7.05)	417.23 (383.02)	0.19 (0.11)
AA (*N* = 13)	115.90 (80.30)	9.08 (6.50)	448.83 (189.46)	0.17 (0.12)
*PDE1C* rs30561			^*^P = 0.038	
CC (*N* = 109)	109.14 (86.83)	10.88 (5.96)	391.37 (312.57)	0.19 (0.12)
CT/TT (*N* = 27)	95.49 (90.43)	9.08 (5.75)	480.47 (528.32)	0.18 (0.10)
*PDE3A* rs11045347				
TT (*N* = 44)	113.23 (87.03)	11.77 (5.87)	479.73 (344.97)	0.19 (0.12)
CT (*N* = 66)	94.61 (82.25)	9.17 (7.16)	417.97 (336.98)	0.19 (0.10)
CC (*N* = 26)	119.81 (120.29)	10.53 (13.05)	359.71 (318.16)	0.17 (0.16)
*PDE4D* rs37576				
TT (*N* = 64)	107.33 (76.83)	10.22 (6.34)	400.30 (376.50)	0.18 (0.10)
CT (*N* = 58)	97.99 (108.67)	10.74 (8.09)	443.23 (326.84)	0.19 (0.12)
CC (*N* = 14)	94.67 (87.59)	8.87 (1.68)	439.89 (203.57)	0.19 (0.10)
*PDE4D* rs6889641	^*^P = 0.028			
GG (*N* = 80)	99.08 (83.60)	10.23 (6.07)	441.74 (350.53)	0.18 (0.11)
AG (*N* = 47)	112.84 (94.90)	10.18 (9.05)	391.21 (283.83)	0.19 (0.13)
AA (*N* = 9)	118.90 (86.22)	13.86 (7.02)	572.18 (338.22)	0.21 (0.10)
*PDE11A* rs2695735				
AA (*N* = 115)	99.58 (88.01)	10.18 (6.93)	416.64 (363.05)	0.19 (0.13)
AG (*N* = 21)	112.70 (86.58)	11.22 (5.67)	480.47 (250.68)	0.19 (0.08)
*SLC28A2* rs11854484				*P* = 0.071
CC (*N* = 113)	104.48 (90.65)	10.72 (70.23)	446.92 (369.36)	0.19 (0.11)
CT/TT (*N* = 23)	98.57 (62.78)	8.82 (4.90)	348.48 (260.21)	0.16 (0.12)

^*a*^ * indicates *P* < 0.05 in univariate linear regression analysis, and ** indicates *P* < 0.05 in the adjusted analysis.

### Association of SNPs and HBV and HIV virological responses

None of the evaluated SNPs were associated with HBV non-suppression. One SNP *SLC28A2* rs11854484 was associated with HIV non-suppression. Four out of 23 (17.39%) *SLC28A2* rs11854484 T carriers had HIV non-suppression compared to 6 out of 113 (5.31%) in those with CC genotype, with adjusted OR of HIV non-suppression of 5.14 (1.14–23.09), *P* = 0.033 (Table 4). Two other SNPs had a trend for association with HIV non-suppression. 18.18% of *ABCC2* rs717620 CT carriers vs 6.40% of CC participants had HIV non-suppression, with an adjusted OR of 6.38 (0.89–45.5) (*P* = 0.065) (Table 4). 14.81% of participants are *PDE1C* rs30561 CT carriers vs 5.50% of participants with CC genotype HIV non-suppression, with adjusted OR of 4.25 (0.79–22.79), *P* = 0.091. When these three SNPs were evaluated together, participants with two or three of these variants were significantly more likely to have HIV non-suppression: 42.86% vs 6.52% in participants carrying one of these variants and 4.82% in participants with none of these variants (*P* = 0.001). The adjusted OR was 48.25 (4.25–547.82) (*P* = 0.0016) for participants with two or three variants compared to those with none of these variants (Table 4).

**TABLE 4:** Association of three SNPs and their combined scores and HIV non-suppression

Gene	SNP	Genotype	*N*, total	*N*, HIV non-suppression	HIV non-suppression (%)	Adjusted OR^*a*^	95% CI	*P*
*SLC28A2*	rs11854484	CC	113	6	5.31%	Reference		
		CT/TT	23	4	17.39%	5.14	1.14–23.09	0.033
*ABCC2*	rs717620	CC	125	8	6.40%	Reference		
		CT	11	2	18.18%	6.38	0.89–45.5	0.065
*PDE1C*	rs30561	CC	109	6	5.50%	Reference		
		CT/TT	27	4	14.81%	4.25	0.79–22.79	0.091
	3-SNP score	Score of 0	83	4	4.82%	Reference		
		Score of 1	46	3	6.52%	1.39	0.21–9.00	0.092
		Score of ≥2	7	3	42.86%	48.25	4.25–547.82	0.0016

^*a*^ Logistic regression adjusted for age, sex, body mass index, and CD4 counts.

## DISCUSSION

In this study, we investigated the association between SNPs in genes encoding for transporters that may be involved in the uptake or efflux of TFV and 3TC and their phosphate anabolites and PDE enzymes with concentrations of TFV-DP and 3TC-TP in PBMCs and DBS. In addition, we examined the relationship between the evaluated SNPs and HBV or HIV non-suppression. We found a significant association between *ABCC2* rs2273697 SNP and 3TC-TP concentrations in PBMCs in both the unadjusted and multivariable analyses that adjusted for age, sex, BMI, and CD4 count. The median 3TC-TP concentration in PBMCs was significantly higher in *ABCC2* rs2273697 variant carriers compared to individuals with the wild type. The *ABCC2* rs17216177 SNP was associated with 3TC-TP concentrations in PBMCs in the univariate analysis but not in the adjusted analysis. The efflux transporter MRP2 (encoded by *ABCC2*) is expressed at important pharmacological barriers, such as the enterocytes, lymphocytes, epithelial cells of proximal tubules in the kidney, and the canalicular membrane of hepatocytes (31). The significant association between *ABCC2* rs2273697 SNP and 3TC-TP concentrations in PBMCs has not been previously reported (to the best of our knowledge), and the mechanism is unknown. The association of *ABCC2* rs2273697 with 3TC-TP concentrations in PBMCs suggests that 3TC and/or its intermediate anabolites may be a substrate of MRP2. Our findings suggest that the carrier of the *ABCC2* rs2273697 variant may have reduced the efflux activity of the transporter, leading to the accumulation of 3TC and/or metabolites in cells. In a case–control study in Korea, the A allele of the non-synonymous polymorphism *ACCC2* c.1249G>A (p.V417I, rs2273697) SNP was associated with adverse neurological drug reactions to carbamazepine due to selectively reduced carbamazepine transport across the cell membrane (32). The clinical importance of this SNP requires further investigations in populations of African and European backgrounds as the minor allele frequency of *ABCC2* rs2273697 SNP is 9.1% in Asians and 18%–20% in Africans and Europeans (33).

In the univariate analysis, *PDE4D* rs6889641 was associated with TFV-DP concentrations in PBMCs, while PDE*1C* rs30561 and *ABCC2* rs3740066 were associated with TFV-DP concentrations in DBS. It is biologically plausible that the *PDE* SNPs could be associated with TDF disposition and intracellular TFV-DP concentrations as TDF is converted to a monoester upon absorption and then to TFV by a PDE upon first pass through the liver (19). Thus, the marginal associations between *PDE4D* rs6889641 and *PDE1C* rs30561 with TFV-DP concentrations should be examined in future studies of TDF pharmacogenetics. The T allele of *ABCC2* rs3740066 was also associated with higher TFV-DP concentrations in DBS in our study. Among healthy Chinese volunteers treated with TAF, participants with the TT genotype in rs3740066 had significantly longer TFV terminal half-life than those with the rs3740066 genotype (34). TFV could be a substrate of ABCC2 and should be investigated. The *ABCC2* 1249G>A rs2273697 SNP was found to be associated with TDF-induced renal proximal tubulopathy in PWH in one study in France (35), but we found no significant association between the SNP and intracellular TFV-DP concentrations in the current study. We also did not find a significant association between *ABCC4* 3463A>G rs1751034 genotype and TFV-DP in PBMCs as previously reported (29). A study of 30 PWH (six black) in the United States on TDF and lopinavir/ritonavir-containing regimen reported 35% higher TFV-DP concentrations in PBMCs in carriers of the *ABCC4* 3463G variant than those with the wild type (29). Unlike the above-mentioned study where participants were on concurrent lopinavir/ritonavir with TDF (29), our study participants were predominantly on dolutegravir or efavirenz-based ART. There is an interaction between TFV and lopinavir/ritonavir that manifests a decrease in TFV renal clearance by 17.5% (36). Thus, drug–drug and drug–gene interactions could have contributed to the differences in findings between our study and that by Kiser et al. (29). A study by Anderson et al. found that the concentrations of 3TC-TP in PBMCs increased by 20% in participants s carrying the *ABCC4* 4131TG or GG genotype than those carrying the TT wild-type genotype (28). The proposed mechanism of this interaction was that the *ABCC4* 4131T>G variant reduces MRP4 expression, thereby decreasing the transport of drugs in renal tubular cells (28). We were unable to confirm the association because the distribution of the *ABCC4* 4131T>G SNP genotypes in our population deviated from HWE and was excluded from the association analysis.

In the analysis of the association between the studied SNPs and virologic response, the *SLC28A2* rs11854484 SNP was associated with HIV non-suppression in the multivariable analyses. There was also an additive effect of the SNP with two other SNPs (*ABCC2* rs717620 and *PDE1C* rs30561) that showed a trend toward HIV non-suppression. To the best of our knowledge, the association of these SNPs with HIV non-suppression has not been previously reported and needs to be confirmed. The S*LC28A2* 124 CT/TT genotype was reported to be an independent predictor of TFV plasma exposure together with estimated creatinine clearance and protease inhibitor coadministration (37). The underlying mechanism that links *SLC28A2* rs11854484 SNP with HIV non-suppression is unknown and does not appear to be due to higher TFV-DP concentrations in those with S*LC28A2* 124 CT/TT genotype. Interestingly, *SLC28A2* rs11854484 correlated with sustained virologic response (SVR) in people with chronic hepatitis C virus (HCV) infection treated with pegylated interferon plus ribavirin where 56.3% of patients with rs11854484 TT (*n* = 32) had SVR compared with 30.8% of patients with rs11854484 CC/CT (*n* = 65), *P* = 0.016 (38). Considering patients infected by HCV-genotype 2/3, the correlation of rs11854484 with SVR was significant as 89.3% of patients with rs11854484 TT (*n* = 28) had SVR compared with 67.3% of patients with rs11854484 CC/CT (*n* = 65), *P* = 0.032 (38). The effect of the T allele on HIV suppression seems to be the opposite for HCV but provides additional evidence that *SLC28A2* rs11854484 may influence antiviral activity. The findings of this study did not confirm our prior report of a significant relationship between *ABCC4* rs11568695 SNP and HBV suppression in Ghanaian individuals with HIV/HBV coinfection (39). The associations of the *ABCC2* rs717620 and *PDE1C* rs30561 with HIV non-suppression did not reach statistical significance, but it had an additive effect when combined with the S*LC28A2* rs11854484 SNP. This approach may be a more powerful way to determine genotype–phenotype associations and needs to be assessed in future studies.

We recognize that our study has several limitations. The baseline CD4 count and HBV DNA levels were not available to include in the multivariate analyses considering the cross-sectional nature of the study. These factors have all been associated with suboptimal HBV DNA suppression in individuals with HIV/HBV coinfection (11, 12, 14, 15). In addition, our sample size was relatively small, and the subgroup of samples with HBV non-suppression is fewer than we initially planned (24), which may explain why some SNPs were significant in unadjusted but were no longer significant in the multivariate analysis. It is possible that a larger study with a larger proportion of samples with unsuppressed HBV DNA may identify additional genetic variants with stronger effects on intracellular drug exposure and antiviral activity than the one implicated in this study. Also, the lack of a significant relationship between the *ABCC2* rs2273697 SNP and HIV non-suppression could have been due to the small number of participants with the variant in the study as we found an association of 3TC-TP in PBMCs and a trend toward HIV suppression (24). Two SNPs in our study, *ABCC4* T4131G (rs3742106) and *ABCC4* rs1059751, deviated from HWE and were excluded from the association analyses. Despite these limitations, we found a consistent association between *ABCC2* rs2273697 SNP and intracellular 3TC-TP concentration in individuals with HIV/HBV coinfection on TDF/3TC-containing ART and identified a combination of genetic factors associated with HIV non-suppression. Further studies are warranted to verify our findings and elucidate the underlying molecular mechanisms of the *ABCC2* SNP and its contribution to the disposition of 3TC. Finally, although participants were adherent as evidenced by high virologic suppression rates, variable adherence in the weeks prior to the study visit could have impacted drug concentrations.
